# Amino Acid Sequence and Structural Comparison of BACE1 and BACE2 Using Evolutionary Trace Method

**DOI:** 10.1155/2014/482463

**Published:** 2014-08-28

**Authors:** Hoda Mirsafian, Adiratna Mat Ripen, Amir Feisal Merican, Saharuddin Bin Mohamad

**Affiliations:** ^1^Institute of Biological Sciences, Faculty of Science, University of Malaya, 50603 Kuala Lumpur, Malaysia; ^2^Allergy and Immunology Research Centre, Institute for Medical Research, Jalan Pahang, 50588 Kuala Lumpur, Malaysia; ^3^Centre of Research for Computational Sciences and Informatics in Biology, Bioindustry, Environment, Agriculture and Healthcare (CRYSTAL), University of Malaya, 50603 Kuala Lumpur, Malaysia

## Abstract

Beta-amyloid precursor protein cleavage enzyme 1 (BACE1) and beta-amyloid precursor protein cleavage enzyme 2 (BACE2), members of aspartyl protease family, are close homologues and have high similarity in their protein crystal structures. However, their enzymatic properties differ leading to disparate clinical consequences. In order to identify the residues that are responsible for such differences, we used evolutionary trace (ET) method to compare the amino acid conservation patterns of BACE1 and BACE2 in several mammalian species. We found that, in BACE1 and BACE2 structures, most of the ligand binding sites are conserved which indicate their enzymatic property of aspartyl protease family members. The other conserved residues are more or less randomly localized in other parts of the structures. Four group-specific residues were identified at the ligand binding site of BACE1 and BACE2. We postulated that these residues would be essential for selectivity of BACE1 and BACE2 biological functions and could be sites of interest for the design of selective inhibitors targeting either BACE1 or BACE2.

## 1. Introduction

Members of aspartyl protease family are known to be associated with some pathological states such as breast cancer and pruritic inflammatory skin disease [1]. Furthermore, beta-amyloid precursor protein cleavage enzyme 1 (BACE1), a member of aspartyl protease family, is known to play an important role in the cellular pathways leading to Alzheimer's disease [2]. Beta-amyloid precursor protein cleavage enzyme 2 (BACE2) is a close homolog to BACE1 with 64% amino acid sequence similarity [3], and orthologs of both human BACE1 and BACE2 genes are present in marmoset, cattle, rabbit, guinea pig, rat, and mouse. Southan and Hancock reported the evolutionary history of BACE1 and BACE2, suggesting that the mammalian BACE1 and BACE2 are purifying selection compared to other classes analyzed [4].

BACE1 is a type 1 transmembrane protein which is highly expressed in brain and pancreas, and it can also be found in other organs at much lower levels [5]. The current concept for Alzheimer's disease development involves the cleavage of amyloid precursor protein (APP) to amyloid beta (A*β*) peptide by BACE1 leading to the preliminary constituent of amyloid plaques in the brains of individuals with the disease [6]. BACE2 can be found in the majority of peripheral tissues with different expression levels, with kidney tissues showing the highest level of expression [7]. Though BACE2 is also expressed in the brain, some reports revealed the protective effect of the enzyme towards Alzheimer's disease, by reducing the pathogenic forms of A*β* being produced within the cells that expressed both BACE1 and BACE2 [8]. BACE1 competes with BACE2 over APP substrate, but their enzymatic activity generates different type of products in which the product of BACE1 but not BACE2 leads to Alzheimer's disease [9]. Furthermore, BACE1 is known as rate-limiting enzyme for A*β* formation leading to Alzheimer's disease, making it a good target for drug design to treat Alzheimer's disease. Since BACE1 activity is known to cause Alzheimer's disease, it is essential to design a selective drug that could specifically target BACE1 over BACE2 [2].

Bioinformatics tools have been widely used for predicting protein functions. Various computational methods for predicting protein structure and functions have been developed. Methods to predict protein functions can be divided into sequence-based method and structure-based method [10]. In this study, evolutionary trace (ET) method which relies on both amino acid sequence and structural information to analyze functional sites of proteins is used because of the advantage of having molecular and structural information in a single analysis [11]. It identifies the amino acid conservation pattern of a group of proteins and transfers the information onto known 3D protein structures. Since amino acid residues that are vital for function or structural stability of a protein tend to be conserved across species, any differences at the functional sites would indicate possible differences for biological activity. Here, we reported the application of the ET method on BACE1 and BACE2 to extract the conservative and distinctive features of both enzymes in terms of molecular sequences and 3D structures that would provide beneficial details for the design of selective drug targeting BACE1 and BACE2.

## 2. Materials and Methods

### 2.1. Dataset

Human BACE1 and BACE2 sequences with UniProt accession numbers P56817 and Q9Y5Z0, respectively, were used as query sequences for BLASTP [12] searches against the UniProt database [13]. Selected BACE1 and BACE2 mammalian sequences with more than 80% identity to their respective query sequence were selected form BLASTP search results.

### 2.2. Evolutionary Trace Analysis

**Figure 1:**
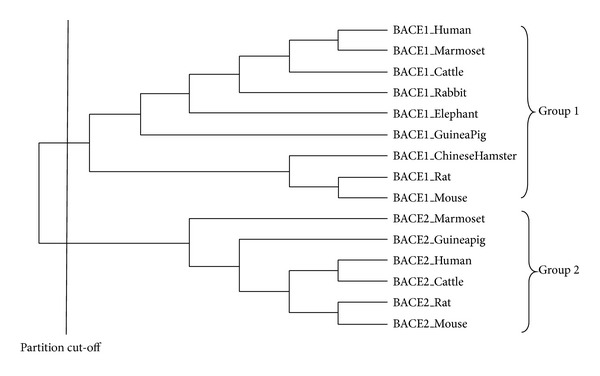
Evolutionary trace based cladogram of selected BACE1 and BACE2 protein sequences from UniProt database. ET partition cut-off divides the phylogenetic tree into Group 1 (BACE1) and Group 2 (BACE2).

Multiple sequence alignment was carried out on the selected sequences using ClustalW [14] and a phylogenetic tree was generated using Neighbor-joining algorithm method and visualized by PhyloDraw [15]. After the phylogenetic tree had been constructed, a vertical cut-off line was generated at the node of the tree which divided the tree into two groups with one group containing all BACE1 sequences while the other was with all BACE2 sequences (Figure 1).

**Figure 2:**
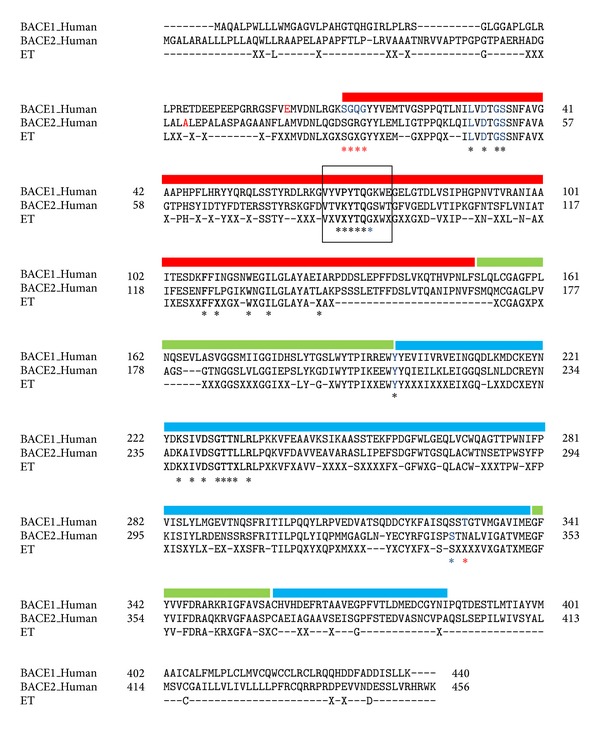
ET sequence compared to the seed sequences of BACE1 (P56817) and BACE2 (Q9Y5Z0). The domains within BACE1 and BACE2 sequences are marked with the coloured thick lines. Red and blue lines are for the N-terminal and C-terminal domains, respectively, whereas the green lines marked the interdomain regions within the structures. Amino acid residues for the flap region are marked within the box. Conserved residues are shown as their specific amino acid in the ET sequences, while group-specific residues are shown as alphabet “X.” Neutral residues are marked as “-” in the ET sequence. The amino acid numbering of BACE1 and BACE2 sequences is according to their 3D structures obtained from PDB file, 1FKN, and 2EWY, respectively. The starting amino acid residues are highlighted in red. Amino acid residues that are involved in the ligand binding sites of BACE1 and BACE2 are marked with asterisk. Red coloured asterisks are binding sites that are only for BACE1, while blue coloured asterisks are the ligand binding sites that are only for BACE2.

Then, the sequences were separated based on groups. Sequences within each group were separately aligned together and consensus sequences were obtained for a particular group. The consensus sequences were classified as neutral, group-specific, and conserved residues. Conserved residues are amino acid residues that are conserved in the multiple sequence alignment while neutral residues are amino acid residues that are not conserved. Group-specific residues are amino acid residues that are conserved within a particular group but differ from other groups. The consensus sequences were then compared and an ET sequence was derived. ET sequences were compared to the query sequences (P56817, Q9Y5Z0). To identify the location of conserved and group-specific amino acid residues, the ET sequence was aligned with the query sequences (Figure 2).

Crystal structures of human BACE1 and BACE2 with the PDB codes 1FKN and 2EWY, respectively, were retrieved from Protein Data Bank (PDB) [16] and visualized by Python based Molecular Viewer software [17]. In order to analyze the ligand binding site of BACE1 and BACE2, only chain A of both 3D structures was selected, and amino acid residues within 5 Å distance from the ligand were defined as ligand binding sites. Ligand binding residues were compared to the ET sequence to identify their trace status and later mapped onto the 3D structure.

## 3. Results and Discussion

We analyzed BACE1 and BACE2 protein sequences from several mammalian species using ET method to identify the functional sites that lead to different functional properties of BACE1 and BACE2. Developed by Lichtarge et al. in 1996, ET method enables the identification of conservation pattern within homologous proteins by comparing both the amino acid sequence and protein crystal structure information [11]. ET method applications were also reported in the identification ligand binding site [18] and the design of a high hydrophobicity laccase [19], an enzyme with industrial importance.

Our ET analysis revealed that 189 amino acid residues were conserved which is 37.7% of human BACE1 and 36.4% of human BACE2. Furthermore, 123 group-specific residues were identified, which comprise 24.5% of human BACE1 and 23.7% of human BACE2.

**Figure 3:**
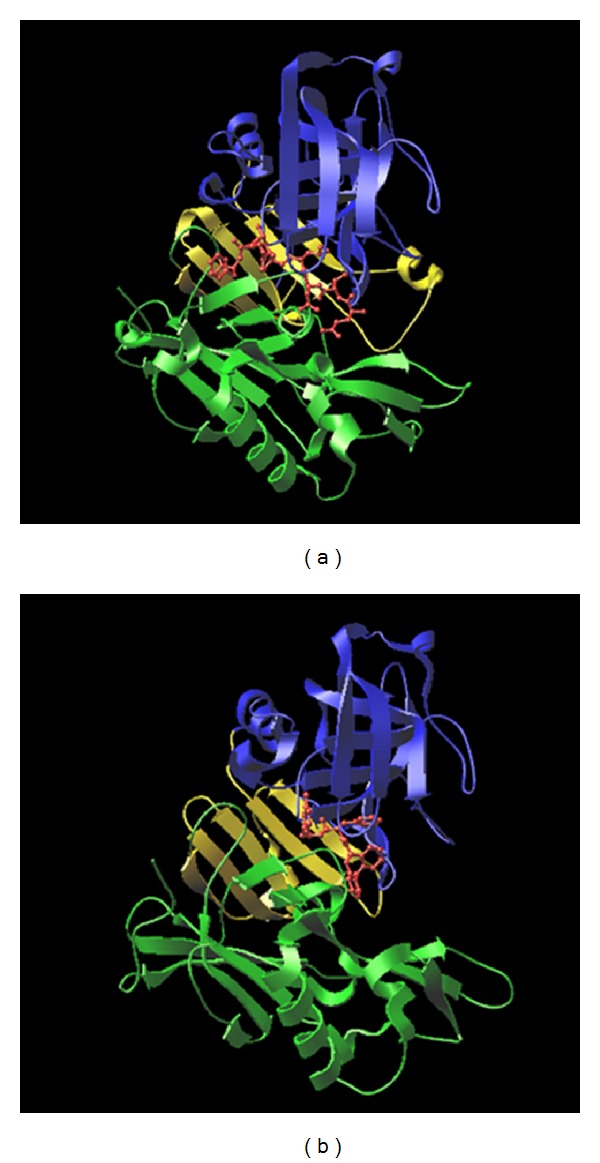
Chain A of crystal structures of BACE1 (a) and BACE2 (b). In both structures, the N-terminal, inter-, and C-terminal domains are coloured blue, yellow, and green, respectively. The ligands are displayed with “Ball and Stick” and red colour.

BACE1 and BACE2 are close homologs that not only share sequence similarity, but also have very similar 3D structure. Overall structures of BACE1 and BACE follow the general fold of aspartyl protease family comprising an N-terminal domain, a C-terminal domain, and an interdomain that connects the N-terminal and C-terminal domains (Figure 3) [9].

**Figure 4:**
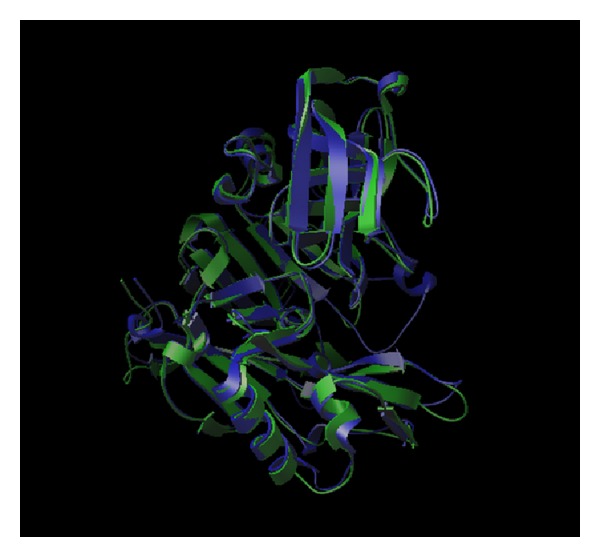
Superimposition of chain A of crystal structures of BACE1 (1FKN) and BACE2 (2EWY), where BACE1 is coloured blue and BACE2 is coloured green.

Superimposition of the crystal structures of BACE1 (1FKN) and BACE2 (2EWY) gave a root-mean-square deviation (RMSD) of 1.46 over 373 C-alpha atoms indicating that the structures of BACE1 and BACE2 are very similar to each other (Figure 4). Although BACE1 and BACE2 have the same biological activity against the APP substrate, their cleavage sites on APP are different, producing different cleavage products which are amyloidogenic for BACE1 and nonamyloidogenic for BACE2.

We defined the ligand binding site as all amino acid residues within 5 Å from the ligand. Twenty-eight of amino acid residues in BACE1 and 24 of amino acid residues in BACE2 were identified as ligand binding site. The ligand binding site residues of BACE1 and BACE2 together with their evolutionary trace status are summarized in Table 1. The entire ligand binding site residues were identified as conserved or group-specific residues and no neutral residues were found in the ligand binding site. This conservation pattern indicates that the active site for both BACE1 and BACE2 is well preserved for the aspartic protease activity. Two catalytic aspartic acid residues which are located in ligand binding site and essential for the enzyme activity are conserved in our analysis (BACE1: Asp32 and Asp228; BACE2: Asp48 and Asp241). However, we identified 4 residues that are group-specific in the ligand binding sites (Table 1). These group-specific residues are Pro70, Ile110, Ile126, and Asn233 of BACE1 substituting Lys86, Leu126, Leu142, and Leu246 of BACE2, respectively. Since we generated a partition cut-off on the phylogenetic tree that differentiates BACE1 and BACE2 into 2 separate groups, we believe that these 4 residues would play a pivotal role in determining the selectivity of enzymatic activity.

**Figure 5:**
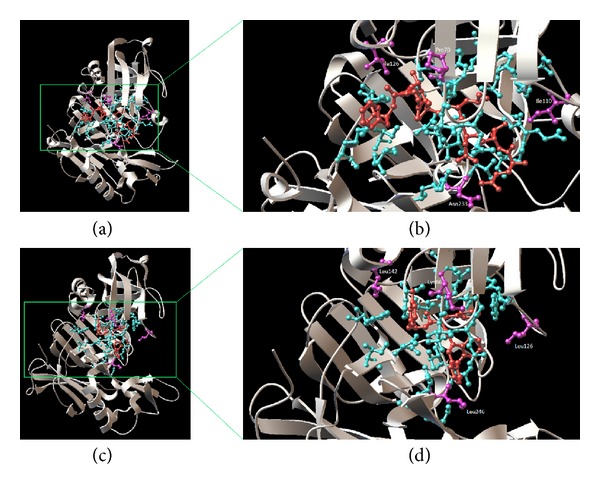
The display of ligand, conserved, and group-specific residues within the ligand binding sits of BACE1 (a, b) and BACE2 (c, d). The ligands are coloured red while conserved and group-specific residues are coloured cyan and purple.

We mapped the ligand binding sites and their ET status onto the chain A of crystal structures of BACE1 (1FKN) and BACE2 (2EWY) (Figure 5). Two out of the 4 group-specific residues identified at the ligand binding site comprise Ile110 and Ile126 for BACE1 substituted with Leu126 and Leu144 for BACE2, respectively. Although these amino acid residues have similar physicochemical properties which are small and hydrophobic, the difference in the orientation of the alkyl group of Ile and Leu residues within the 3D structure of the protein may affect the binding selectivity of the ligand to BACE1 and BACE2.

Members of aspartyl protease family are known to have a loop structure that covers the active site of enzyme upon binding with the ligand. This structure is known as the flap region, and it is flexible and can adopt different structural conformations upon binding with the substrate. It is assumed that this structure will shield the active site from the solvent [20]. The flap region in BACE1 and BACE2 involves residues Val67 to Glu77 and Val83 to Thr93, respectively (Figure 2). In BACE1, Tyr71 of the flap region is known to play an important role in the conformational flexibility of flap region upon binding to ligand (reviewed in [21]). The amino acid sequences of flap in BACE1 and BACE2 are highly conserved. As shown in Figure 2, five residues of the flap were identified at the ligand binding site. Our analysis based on the ET method using a broader range of mammalian BACE1 and BACE2 sequences revealed that, in this region, Pro70 of BACE1 is substituted with Lys86 of BACE2. Since the cyclic property of proline residue may affect the flexibility of the flap region, this group-specific residue could be a crucial site that would lead to selectivity in the enzymatic activity of BACE1 and BACE2.

The last group-specific residue at the ligand binding site is Asn233 of BACE1 substituted with Leu246 of BACE2. The observation indicated that, in BACE2, the binding site at this region is more hydrophobic than the one in BACE1. This difference can be exploited for the design of a selective drug targeting either BACE1 or BACE2.

Designing a drug that could selectively inhibit BACE1 could avoid the unwanted side-effects of also inhibiting other aspartic protease family members including BACE2. BACE1 and BACE2 are close homologs and they are competing against each other for the same substrate (A*β*). As a result, the design of selective BACE1 or BACE2 inhibitors is rather challenging. Analyzing the sequences of a group of selected BACE1 and BACE2 proteins and mapping the details on known 3D structure reveal the differences between these two groups that would be beneficial for the selective drug design.

## 4. Conclusion

ET analysis on amino acid sequences and protein structures of BACE1 and BACE2 from several mammalian species enabled us to identify the distinctive features of BACE1 and BACE2 amino acid sequences. Mapping the ET analysis onto a known 3D structure of BACE1 and BACE2 revealed that their active sites are well conserved. Four group-specific residues were identified in the ligand binding sites of BACE1 and BACE2. The residues are Pro70, Ile110, Ile126, and Asn233 of BACE1 substituting Lys86, Leu126, Leu142, and Leu246 of BACE2, respectively. These group-specific residues would be the reason for cleavage site selectivity in BACE1 and BACE2 biological function and would be the potential residues for the design of selective and specific inhibitors targeting either BACE1 or BACE2.

## Figures and Tables

**Table 1:** Amino acid residues at the ligand binding sites of BACE1 and BACE2. The amino acid residue numbering is according to their respective 3D structure (1FNK and 2EWY).

BACE1		BACE2	
Amino acid residues	Trace status	Amino acid residues	Trace status
Ser10	Conserved	∗∗	—
Gly11	Conserved	∗∗	—
Gln12	Conserved	∗∗	—
Gly13	Conserved	∗∗	—
Leu 30	Conserved	Leu46	Conserved
Asp32	Conserved	Asp48	Conserved
GLy34	Conserved	Gly50	Conserved
Ser35	Conserved	Ser51	Conserved
Val69	Conserved	Val85	Conserved
Pro70	Group-specific	Lys86	Group-specific
Tyr71	Conserved	Tyr87	Conserved
Thr72	Conserved	Thr88	Conserved
Gln73	Conserved	Gln89	Conserved
∗	—	Gln90	Conserved
Phe108	Conserved	Phe124	Conserved
Ile110	Group-specific	Leu126	Group-specific
Trp115	Conserved	Trp131	Conserved
Ile118	Conserved	Ile134	Conserved
Ile126	Group-specific	Leu142	Group-specific
Tyr198	Conserved	Tyr211	Conserved
Lys224	Conserved	∗∗	—
Ile226	Conserved	Ile239	Conserved
ASP228	Conserved	Asp241	Conserved
Gly230	Conserved	Gly243	Conserved
Thr231	Conserved	Thr244	Conserved
Thr232	Conserved	Thr245	Conserved
Asn233	Group-specific	Leu246	Group-specific
Arg235	Conserved	Arg248	Conserved
Thr329	Conserved	∗∗	—
∗	—	Ser337	Conserved

∗Ligand binding site not identified in BACE1.

∗∗Ligand binding site not identified in BACE2.
